# Congenital Malformations among Babies Born Following Letrozole or Clomiphene for Infertility Treatment

**DOI:** 10.1371/journal.pone.0108219

**Published:** 2014-10-01

**Authors:** Sunita Sharma, Sanghamitra Ghosh, Soma Singh, Astha Chakravarty, Ashalatha Ganesh, Shweta Rajani, B. N. Chakravarty

**Affiliations:** 1 Institute of Reprooductive Medicine, Kolkata, West Bengal, India; 2 School of Medical Science and Technology, Indian Institute of Technology, Kharagpur, India; Medical College of Wisconsin, United States of America

## Abstract

**Context:**

Clomiphene citrate (CC) is the first line drug for ovulation induction but because of its peripheral antiestrogenic effect, letrozole was introduced as the 2^nd^ line drug. It lacks the peripheral antiestrogenic effect and is associated with similar or even higher pregnancy rates. Since letrozole is a drug for breast cancer, its use for the purpose of ovulation induction became controversial in the light of studies indicating an increased incidence of congenital malformations.

**Aims:**

To evaluate and compare the incidence of congenital malformations among offsprings of infertile couples who conceived naturally or with clomiphene citrate or letrozole treatment.

**Settings and Design:**

A retrospective cohort study done at a tertiary infertility centre.

**Methods and Material:**

A total of 623 children born to infertile women who conceived naturally or following clomiphene citrate or letrozole treatment were included in this study. Subjects were sorted out from medical files of both mother and newborn and follow up study was done based on the information provided by parents through telephonic conversations. Babies with suspected anomaly were called and examined by specialists for the presence of major and minor congenital malformations. Other outcomes like multiple pregnancy rate and birth weight were also studied.

**Results:**

Overall, congenital malformations, chromosomal abnormalities were found in 5 out of 171 (2.9%) babies in natural conception group and 5 out of 201 babies in the letrozole group (2.5%) and in 10 of 251 babies in the CC group (3.9%).

**Conclusions:**

There was no significant difference in the overall rate of congenital malformations among children born to mothers who conceived naturally or after letrozole or CC treatment.

**Key Messages:**

Congenital malformations have been found to be comparable following natural conception, letrozole and clomiphene citrate. Thus, the undue fear against letrozole may be uncalled for.

## Introduction

Letrozole is a third-generation selective aromatase inhibitor that inhibits the production of estrogen from androstenedione and testosterone substrates. It is a widely recommended drug for the treatment of postmenopausal breast cancer and recently it has proven its role as an effective agent for ovulation induction [1]. Letrozole has several distinct advantages over CC. Clomiphene has both estrogen agonistic and antagonistic properties. CC causes depletion of hypothalamic estrogen receptors leading to increased GnRH secretion, thereby increasing pituitary gonadotropin release and ovarian activity [2]. Though high ovulation rate (60–90%) make it an attractive therapy, the pregnancy rate of 10 to 20% [3] is disappointing. Sub-optimal pregnancy rates with CC have been attributed to its peripheral anti-estrogenic effects, mainly on the endometrium and the cervical mucus [4] or its interference with the functioning of the corpus luteum. Gonadotropins have been used in cases of clomiphene failure but these are very expensive and can lead to hyperstimulation, thus need close supervision and monitoring. Pregnancy outcome with letrozole is comparable to gonadotropin but it is cheaper and devoid of complications of gonadotropin [4], [5].

Concerns have been raised regarding the use of letrozole for ovulation induction, as it might interrupt the normal aromatase function in tissues during early fetal development and can be potentially teratogenic.

This issue was raised at abstract presentation at the 2005 Annual Meeting of the American Society for Reproductive Medicine in which authors reported that the use of letrozole for infertility treatment might be associated with a higher risk of congenital cardiac and bone malformations in the newborns [6]. Following this presentation in late 2005, Novartis Pharmaceuticals, the Swiss company that developed letrozole for treatment of breast cancer, issued a warning to infertility clinics asserting that the company does not advocate the use of this medication for infertility treatment.

Letrozole was being used in India for infertility treatment mainly as a second line drug after CC failure. It is at least as effective as CC and probably more cost effective, second-line option after CC failure in comparison to gonadotropins [7]–[9], especially in a developing country like India. In October 2011 the Ministry of Health and Family Welfare, India issued a directive to suspend the use of letrozole in infertile women with immediate effect citing concerns regarding its safety. We, therefore undertook this study to compare the rate of congenital malformation in the babies of infertile couples who conceived naturally or following administration of letrozole or CC and to the best of our knowledge, it is the first study of such kind in the Asian subcontinent.

## Subjects and Methods

The present study is a retrospective study conducted at a tertiary level infertility centre to identify and follow up babies born between January 2007 and December 2011 to infertile couples, who conceived naturally or following administration of either CC or letrozole. The study was approved by the ethical committee of the institute (Ethical Committee, Institute of Reproductive Medicine). All patients consulting at the institute sign a consent form allowing the use of their information except personal identification for the purpose of research or publication. Similarly, on admission for delivery, parents sign a consent form allowing the use of their and their newborn’s medical information for research purpose. It was a retrospective study and data was obtained from the medical files of mothers and newborns.

Infertile women in the age group of 21–35 years who conceived naturally or undergoing ovulation induction or augmentation for timed- intercourse or intrauterine insemination who received either letrozole (5 mg) or CC (100 mg) orally daily for 5 days from day 3 to 7 of the cycles were included in this study. Women who conceived spontaneously during investigations like HSG, Laproscopy or those who conceived while waiting for some procedure were included in the natural conception group. All the groups were carefully scrutinized for the presence of risk factors for congenital malformation like history of congenital/chromosomal anomaly in the previous pregnancies or recurrent pregnancy loss, family history of birth defects, presence of medical disorders and concomitant use of other drugs, smoking, or alcohol during pregnancy or occupational exposure to endocrine disruptors/radiation.

**Figure 1:**
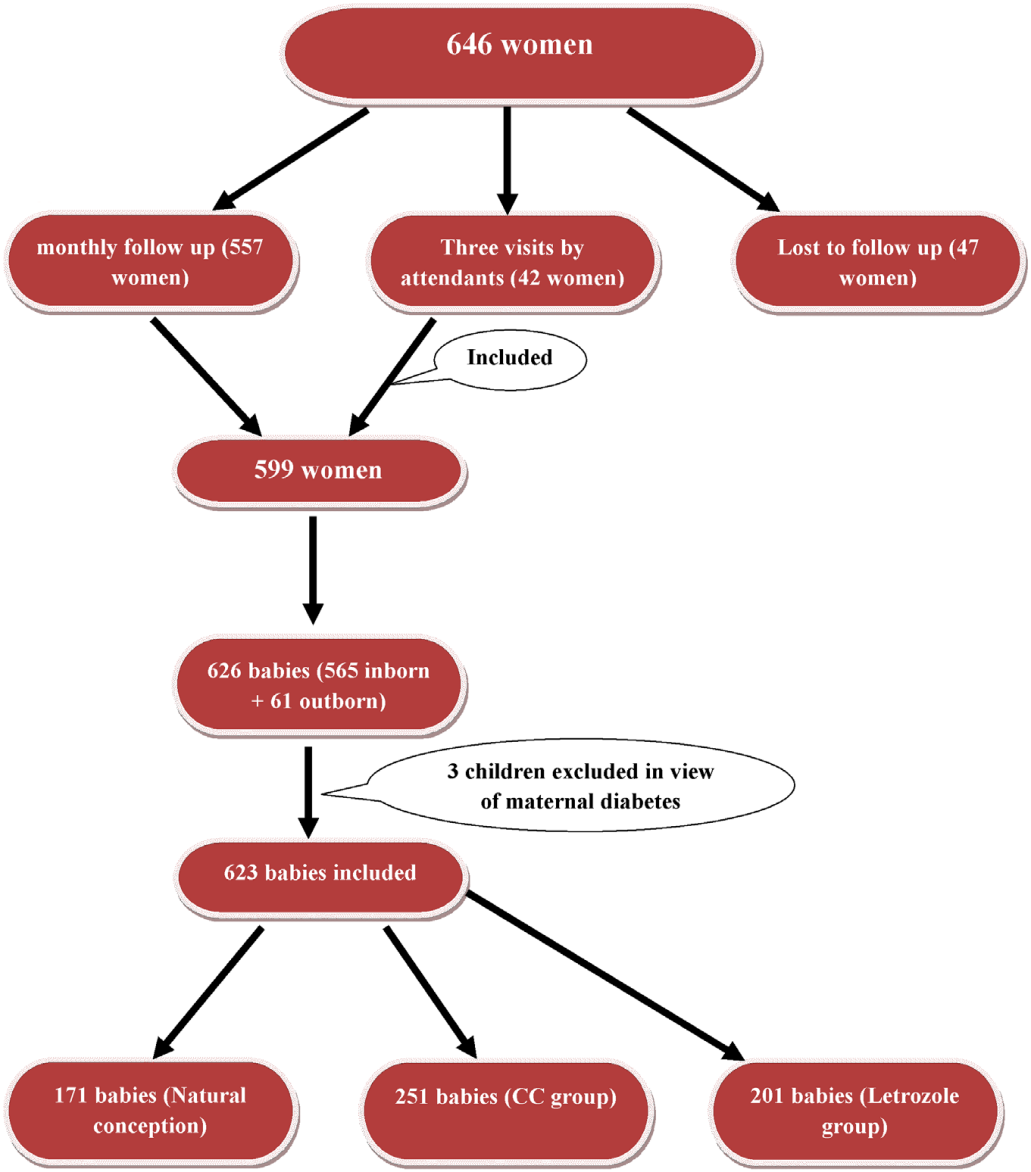
Flowchart showing the women and children included in the study.

As a routine, following conception, women follow up for antenatal check up at our institute. Pregnant women who cannot come for regular follow up are advised to visit at least thrice for antenatal check-up and at least once at 6 weeks post conception. These visits include an initial scan at 6 weeks for confirmation of number of sacs and viability of pregnancy, second visit at 18 weeks for triple marker screen and ultrasonography to rule out anomalies, third visit at 36 weeks. In the present study, out of 599 women, 557 managed to come for a regular monthly follow up and for the remaining 42 women, patient’s attendants followed up with the antenatal reports thrice as mentioned above (Figure 1).

In the postnatal visit, the obstetrician routinely examines women and the babies are thoroughly examined by a paediatrician. Electronic records are maintained for all the patients till their postnatal check up.

**Figure 2:**
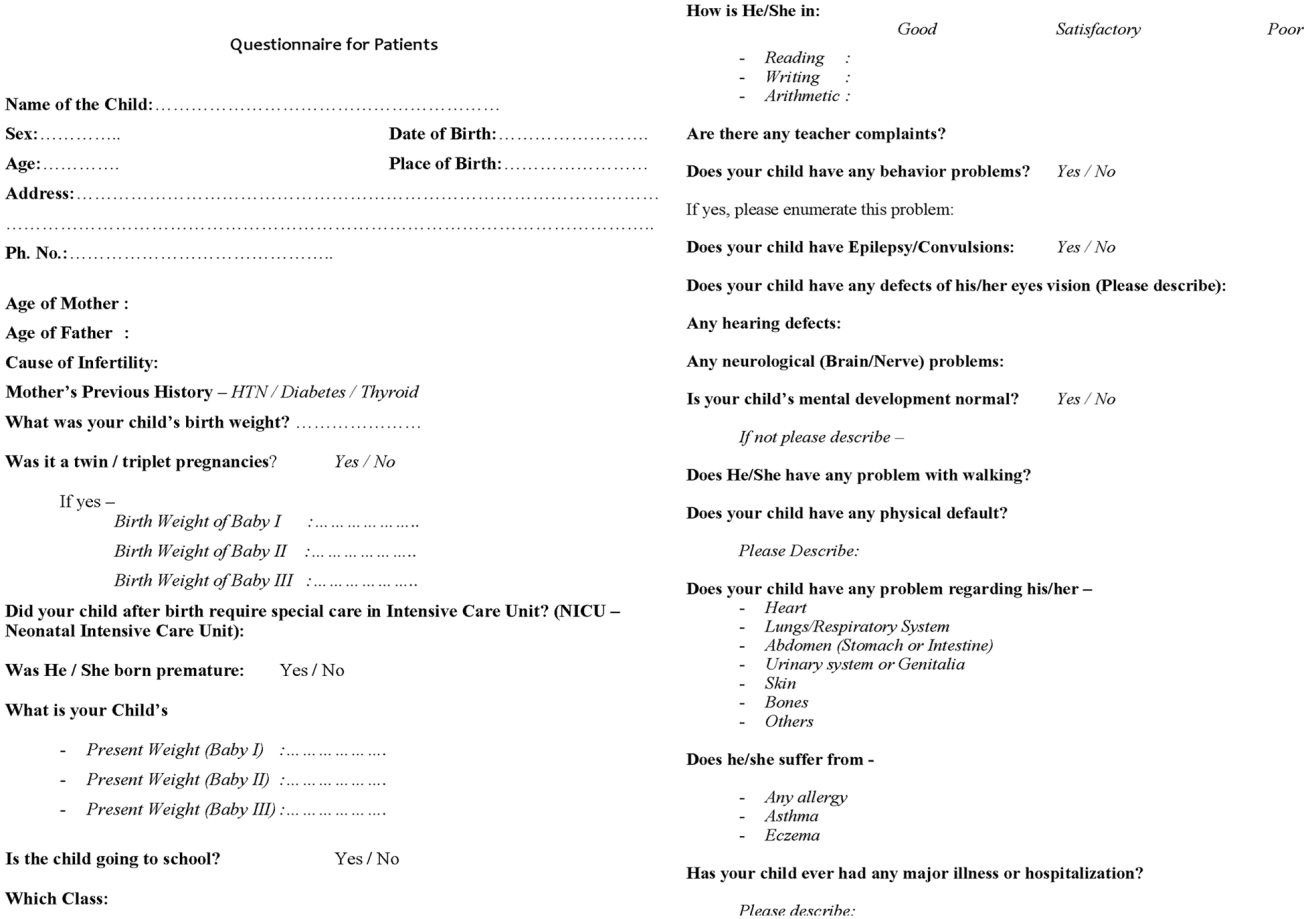
Questionnaire for telephonic follow up of newborns born after letrozole/clomiphene treatment.

For the purpose of this study, babies born both in and outside the institute were included. After an informed consent from the concerned parties, the details of demographic profile, perinatal event and pregnancy outcome were obtained from the medical files of both mother and baby which were later verified (with the parents) by telephone calls. All telephonic conversations were made by trained nurses who filled up a questionnaire provided in Figure 2. Parents were also enquired about any adverse postnatal event affecting the babies after discharge from the hospital, congenital malformations, developmental milestones and wellbeing till their present age with help of a questionnaire prepared for the purpose (Figure 2). Complete data collection was possible only for 626 babies, out of which 61 babies delivered outside (Figure 1). For these 61 children, information regarding the place of delivery (institutional/home), mode of delivery, perinatal events and whether the newborn was examined by a paediatrician was collected. Babies with congenital malformation/delayed milestones/suspected anomaly were called and examined by pediatricians at the institute or referred to sub-specialists for confirmation and management of the anomaly. Data could not be retrieved for 47 pregnancies (Figure 1) i.e. 10 in natural conception group, 16 in letrozole group and 21 in clomiphene group as they had delivered outside the institute and their contact numbers had changed or were not functional and who did not respond to the postal letters sent from the institute. These children were excluded for the purpose of this study. The definition of congenital malformation, deformations and chromosomal abnormalities as stated in Chapter XVII, ICD-10 World Health Organization, International Statistical Classification of Diseases and Related Health Problems was used for the study purpose.^ 10^According to WHO, Congenital anomalies are also known as birth defects, congenital disorders or congenital malformations. Congenital anomalies can be defined as structural or functional anomalies, including metabolic disorders, which are present at the time of birth.

### Statistical analysis

The proportion of congenital malformations between the different study groups were compared by Yate’s corrected Chi square test. Anomaly risk was quantified by univariate odds ratio with 95% CI. P<0.05 was considered statistically significant. Graph Pad Prism version 5 (San Diego, California: Graph Pad soft ware Inc, 2007) software was used for statistical analysis. Unpaired *t* tests were used to compare the mean centiles between each group. Age and birth weight between different groups were compared by student t test.

## Results

In our study, we obtained complete data for a total of 626 babies born to mothers who conceived either naturally or following CC or letrozole from Jan 2007 to Dec 2011. Age-wise distribution of the children in both the groups is shown in Table 1. The number of children >4 years were only 4 in the letrozole group as letrozole was not quite popular as an ovulation induction agent in 2007 and was being used sparingly.

**Table 1:** Age–wise distribution of children.

Age in years	Letrozole	Clomiphene	Naturalconception
	(%)	(%)	(%)
>4	04 (1.99%)	65 (21.11%)	39 (22.80%)
3–4	45 (22.38%)	48 (19.12%)	35 (20.46%)
2–3	39 (19.40%)	53 (21.11%)	37 (21.63%)
1–2	42 (20.89%)	35 (13.94%)	33 (19.29%)
<1	71 (35.32%)	50 (19.92%)	27 (15.78%)
Total	201	251	171

Age of the mothers in the two groups was comparable (Table 2). 171 babies from 171 pregnancies in natural conception group, 201 babies from 192 pregnancies in letrozole group (including 9 sets of twins) and 251 babies from 236 pregnancies in CC group (including 15 pair of twins) were included. Rate of twins was 4.48% in letrozole group and 6.35% in CC group. There were no twins in natural conception group. There were no higher order pregnancies. The mean birth weight of babies in the CC group was significantly lower than those in the letrozole group (2.50±0.42 *vs* 2.59±0.41, p<0.02) after excluding twin births, as shown in Table 2. The mean birth weight of babies was comparable in natural conception group and letrozole group.

**Table 2:** Birth weight of the newborns, incidence of multiple births and age of the mother who conceived following natural conception/letrozole/CC.

Characters	Naturalconception	Letrozole	Clomiphene	P value
No. of newborns	171	201	251	
Twins	none	9	15	NS
Age of mother(years)	29.3±3.85	29.83±3.73	29.84±4.05	NS
Birth weight ofsingleton newborn (kg)	2.61±0.56^a^	2.59±0.41^b^	2.50±0.42^c^	0.02

Values in different columns in superscripts differ significantly. ^a^ *vs.* ^c^: p<0.02; ^b^ *vs*. ^c^: p<0.02.

Structural malformations and chromosomal abnormalities were found in 5 out of 171 (2.9%) babies in natural conception group, 5 out of 201 (2.5%) babies in the letrozole group and in 10 of 251 (3.9%) babies in the CC group (Table 3). One out of 171 babies (0.58%) in the natural conception group was diagnosed with ventricular septal defect (VSD), one out of 201 babies (0.49%) in the letrozole group was diagnosed with congenital heart disease (combined ventricular and atrial septal defect). Three babies in CC group had congenital heart disease, out of which 2 babies had patent ductus arteriosus and one had total anomalous venous connection. Out of these three, two babies were excluded from the analysis, as the mothers of these babies were diabetic on insulin, which is a high risk factor for congenital heart disease. Second commonest anomaly amongst the babies born after CC treatment was hypospadias–3 out of 251 (1.2%) as compared to none in natural conception group and letrozole group. One child in the clomiphene group had bilateral CTEV; this too was excluded from our analysis, as it was born to a diabetic mother. So, in all, 3 children in the CC group were excluded on account of diabetes in the mothers, therefore a total of 251 babies were included in the CC group. In the CC group, one child each was diagnosed with duplication of urethra, cleft lip & palate, inguinal hernia and neural tube defect. In addition, there were two babies with Down’s syndrome in clomiphene group (Table 3).

**Table 3:** Congenital anomalies in different groups.

Anomalies			
	Natural conception	Letrozole	Clomiphene
Cardiac	1 (VSD)*	1 (VSD*+ASD†)	1 (TAPVC§)
Musculoskeletal	2 (1CTEV‡, 1Polydactyly)	1 (CTEV‡)	
Genitourinary system	none	none	4 (3Hypospadias, 1duplication of urethra)
Digestive system	1 (Imperforate anus)	1 (Paraumblical Hernia)	1 (Inguinal Hernia)
Nervous system	none	none	1 (NTD)*
Cleft lip/cleft palate	1	none	1
Ear	none	1 (Congenital deafness)	None
Chromosomal anomalies	none	none	2
Albinism	none	1	none

*Note:* *- Ventricular septal defect.

† - Atrial septal defect.

‡ - Congenital Talipes equino varus.

§ - Total anomalous pulmonary venous connection.

*- Neural tube defect.

Malformations in letrozole group were paraumbilical hernia, congenital deafness, CTEV and albinism in one child each.

There were no malformations in the twins in either group. Other four malformations in natural conception group were CTEV (Congenital talipus equino varus), cleft lip, imperforate anus and polydactyly. No significant differences existed in the overall prevalence of congenital malformations in all three groups (P<0.648) (Table 4).

**Table 4:** Comparison of Congenital Malformations between Different Groups.

	Overall Congenitalmalformation OR*(95% CI†)	Structural MalformationsOR (95% CI)	Chromosomal AnomaliesOR (95% CI)
Letrozole vs Natural conception	1.181 (0.336–4.150)	1.181 (0.336–4.150)	
CC vs Natural conception	0.726 (0.244–2.163)	0.915 (0.294–2.846)	0.291 (0.014–6.103)
CC vs Letrozole	0.614 (0.207–1.829)	0.775 (0.249–2.407)	0.248 (0.018–5.191)
Chi-square (Yate’s corrected)	P<0.648	P<0.907	P<0.226

OR - Odds Ratio.

† CI - Confidence Interval.

When analysis of congenital malformations and chromosomal abnormalities was done separately, no significant difference was found. (P<0.648, P<0.226 respectively) (Table 4).

Compared to babies born after natural conception, CC and letrozole babies had comparable prevalence of overall congenital malformations with Odds ratios between 1.18 and 0.726 (Table 4). We saw a similar trend of prevalence of overall congenital malformation when analysis was done between CC and letrozole with Odds ratio 0.614 (Table 4).

## Discussion

Letrozole has been used for ovarian stimulation since the late 1990s. Studies have shown that it is an effective oral agent for this purpose. It has no significant active metabolites and has a half-life of approximately 45 hours (range 30–60 hours) and should be cleared from the body completely by the time of embryo implantation when used in early follicular phase, for the purpose of ovulation induction.

The study presented by Biljan et al (2005) at ASRM meeting in which they compared 130 letrozole pregnancies with over 36,000 low risk spontaneous pregnancies, reported that letrozole might increase the risk of cardiac and bone anomalies in the newborns [6] however, the overall rate of major malformations did not differ between the two groups. This study, which brought letrozole into disrepute, had many flaws. First and foremost being the heterogeneity between the two groups. Study group included infertile population who by virtue of their infertility are at high risk for having babies with congenital malformations both after spontaneous conception or following infertility treatment [11]. This high-risk population was compared with a low-risk fertile population who had spontaneous conception. Secondly, age of the control population at a standard hospital would likely include a high percentage of younger women (mean age: 30.5 years) who, by nature have a lesser chance of having a malformed baby than a study population of older age (mean age: 35.2 yrs in letrozole group). Thirdly, the reported malformation rate of 1.8% in the “control” population in this study appears to be low as the rate of birth defects in the general population is 3% for major malformations and 6% when minor malformations were included [12]. It is probably because babies with major abnormalities diagnosed on prenatal ultrasound were delivered at a tertiary care hospital rather than a community hospital leading to under-reporting of defects in the “control” population. Moreover, multi-fetal pregnancy rate was higher in the study group, and it is well known that congenital malformations are more common in multiple births than in singletons. Lastly, there was a huge disparity between the sizes of the two study groups (130 vs 36,000). Thus, comparing two unmatched populations makes the study design as well as its results questionable.

Our work focused on comparing the rate of congenital malformation between three groups of infertile women conceiving either naturally or with CC or letrozole. Women of like demographic profile were studied thereby representing a true controlled study. In our study, data of only 4 letrozole babies could be retrieved for the year 2007 (Table 1), because it was being prescribed sparingly at that time and its usage increased in the subsequent years after approval from the drug controller of India. In our study overall rate of congenital malformation is non-significantly higher in natural conception group and CC group compared to letrozole group. The incidence of congenital heart disease among children born following natural conception is comparable to that with letrozole group. Three babies of CC group with congenital heart disease were excluded as they were born to diabetic mothers.

Our results are comparable with that of Tulandi et al, a large retrospective multicentre study done on 911 babies born after ovulation induction with either letrozole (n = 514) or CC (n = 397). In their study, the congenital malformations and chromosomal abnormalities were comparable between the two groups (2.4% in the letrozole group; 4.8% in the CC group) but the rate of all congenital cardiac anomalies were significantly higher (P = 0.02) in the CC group (1.8%) compared to the letrozole group (0.2%) [13]. A recent study looking at birth defects with assisted reproduction also found an increased risk for birth defects in babies born to mothers using CC. After adjusting for confounding factors the odds ratio for CC use and any birth defect was 3.19 (1.32–7.69) [14].

Forman et al, observed lower malformation rate in letrozole group (0%) compared with CC group (2.6%) or spontaneous conception (3.2%) [15]. In a randomized study by Badawy et al comparing 129 deliveries each in CC, letrozole and spontaneous pregnancy groups, authors reported the similar malformation rate in each group [16]. They reported one case of complete cleft palate and one case of major congenital heart problem in the letrozole group and 2 cases of talipus equinovarus in CC and spontaneous pregnancy group. In our study, three cases of hypospadias were found in CC group, out of which 2 had severe (penoscrotal) variety. Our finding is similar to the study done by Meijer et al., who reported high association (OR = 6.08) of severe hypospadias with clomiphene usage [17]. In contrast, Sorenson et.al in their study didn’t find any increased risk of hypospadias with clomiphene [18].

Besides congenital malformations, we also studied mean birth weight and multiple pregnancy rates in all three groups. Mean birth weight was found to be significantly lower in CC group. Our results are similar to study by Forman et al who also reported lower birth weight in CC group compared to the letrozole group, after excluding twin pregnancies [15]. However, other workers have not reported any difference in the birth weight among letrozole, CC and spontaneous pregnancies [16]. It is well established that letrozole diminishes the incidence of multiple gestations because of monofollicular development. We also observed a higher prevalence of twins in CC group than in the letrozole group though the difference was not statistically significant.

Research in animals has shown that letrozole may be teratogenic when used during pregnancy but it does not have any adverse effects when used as an ovulation induction (OI) drug. A study done by Luthra et al (2003) showed that when aromatase-overexpressing mice were treated with high doses of letrozole for 6 weeks and allowed to conceive 2 weeks later, there was no difference between treated and control animals in terms of litter size, birth weight, and anomalies [19]. In another study done at our institute, superovulation in mice with letrozole was shown not to increase the risk to spindle assembly and blastocyst formation in oocytes as evidenced by the birefringent characteristics of the meiotic spindle and preimplantational development of the embryos using Polscope imaging. Study concluded that the risk of aneuploidies or chromosomal defects appears to be low with letrozole [20]. However, regarding its use during pregnancy, animal data has suggested that gestational exposure of letrozole was associated with embryo and fetal toxicity at a concentration much lower than 1% of the human dose [21], [22]. Tiboni et al (2008) observed that exposing rats to letrozole at a dose lower than the recommended human therapeutic dose during pregnancy resulted in a marked increase in intrauterine lethality. The authors concluded that letrozole should not necessarily be regarded as a safe agent for OI although it may not adversely affect morphogenesis when administered before fertilization [23].

A population-based multicenter case control study of major birth defects found an increase in cardiac malformations in the pregnancies following CC [24]. In a review article by Casper et al letrozole seems to be atleast as effective as CC for induction of ovulation and live birth, with some potential advantages over CC [25]. Ongoing, large randomized multicenter studies by clinics in the National Institute of Child Health and Human Development (NICHD) Reproductive Medicine Network are underway that could potentially provide definitive evidence of the efficacy and safety of Letrozole compared with CC for infertility treatment [26].

Regarding the limitation of our study, 47 children were born outside our institute so we had to rely on the information provided by their parents to rule out the presence of anomaly. Moreover, our study is a retrospective study with a relatively small study group; we suggest a large multicentre prospective trial before letrozole is phased out, which can be a valuable addition to the armamentarium of ovulation inducing agents.
